# A Regenerative Immunoaffinity Layer Based on the Outer Membrane of Z-Domains Autodisplaying *E. coli* for Immunoassays and Immunosensors

**DOI:** 10.3390/s18114030

**Published:** 2018-11-19

**Authors:** Daseul Jeon, Jae-Chul Pyun, Joachim Jose, Min Park

**Affiliations:** 1 Cooperative course of Nano-Medical Device Engineering, Hallym University, Chuncheon 24252, Gangwon-do, Korea; M18060@hallym.ac.kr; 2 Department of Materials Science and Engineering, Yonsei University, Seoul 03722, Korea; jcpyun@yonsei.ac.kr; 3 Institute of Pharmaceutical and Medical Chemistry, Westfälische Wilhelms-Universität, 48149 Münster, Germany; joachim.jose@uni-muenster.de; 4 Integrative Materials Research Institute, Hallym University, Chuncheon 24252, Gangwon-do, Korea; 5 Department of Materials Science and Engineering, Hallym University, Chuncheon 24252, Gangwon-do, Korea

**Keywords:** autodisplay, Z-domains, immunoaffinity layer, regeneration, immunosensor, SPR biosensor

## Abstract

Through orientation control of antibodies, Z-domains autodisplaying *Escherichia coli* outer cell membrane (OM) may be utilized to improve the sensitivity and limit of detection (LOD) of immunoassays and immunosensors. A regenerative immunoaffinity layer based on Z-domains autodisplaying *E. coli* OM was developed for the surface plasmon resonance (SPR) biosensor. Regeneration conditions for the Z-domains autodisplaying *E. coli* OM-based immunoassays and immunosensors were optimized by varying pH and detergent concentration. An *E. coli* cell-based HRP immunoassay was tested and validated in three sequential regenerative immunoassays under optimal conditions. The OM of Z-domains autodisplaying *E. coli* was isolated and coated on the two-dimensional substrate (microplate). The OM-based HRP immunoassay was tested and validated in four regenerative immunoassays. This regenerative OM layer was applied to the SPR biosensor. Z-domains autodisplaying OM layered onto the gold surface of SPR biosensors was developed, and the OM-based regenerative immunoaffinity layer with orientation control was tested using CRP analyte. The SPR biosensor regenerative immunoaffinity layer demonstrated that CRP biosensing was repeated for five regeneration cycles with less than 2% signal difference. Therefore, the newly developed regenerative immunoaffinity layer with antibody orientation control may improve biosensing sensitivity and reduce the cost of medical diagnosis.

## 1. Introduction

Biosensors are composed of three major components; a molecular recognition layer, a transducer, and a signal-generating component [1,2]. Biosensors that comprise a molecular recognition layer which uses specific antigen-antibody binding are classified as immunosensors. Immunosensors immobilize target analytes on transducers through the immunoaffinity layer [3]. Antibodies, which bind to specific target antigens, are “Y”-shaped proteins comprising three branches. Antibody binding sites are localized at the tips of the two branches, called the Fab region, and the base area which does not possess antigen binding properties is called the Fc region [4]. In antibodies which are randomly immobilized onto a two-dimensional substrate, less than 20% of antigen binding sites are exposed [5]. Such limited exposure of antigen-binding sites is associated with decreased sensitivity of immunosensors. To overcome this limitation, improvement of immunosensor sensitivity via antibody orientation control is being explored [5,6,7,8,9]. In particular, antibody orientation control via modification of antibody structure is being studied. The use of protein A which binds to the Fc region of human, rabbit, and mouse antibodies to change structural characteristics of these antibodies is a fine case in point [10,11,12,13]. Protein A is a membrane-bound protein derived from *Staphylococcus aureus*, which consists of 5 domains [14,15]. Among these, the B-domain, which has antibody-binding properties, has been widely studied. The Z-domain is an engineered analog of the B-domain which has been used for antibody orientation control and purification due to its binding specificity to the Fc region [16,17].

In previous studies, we expressed Z-domains on the outer membrane (OM) of *E. coli* using the autodisplay technique. Autodisplay is a surface display technology that expresses target proteins on the OM of Gram-negative bacteria through the fusion of autotransporters, such as adhesin, involved in diffuse adherence (AIDA-I) [18,19]. When a target protein is autodisplayed, its N-terminal is connected by a signal peptide, and its C-terminal is connected with a linker and β-barrel. The signal peptide binds to the inner membrane allowing the target protein, linker, and β-barrel to pass through the inner membrane into the periplasm. The autodisplayed protein can anchor onto the bacterial OM β-barrel via a C-terminal, immobilizing the type Va secretion mechanism without any artificial treatment [20,21]. Various proteins, such as Z-domain, adrenodixin, cytochrome P450, esterase, and others, have been expressed via autodisplay technology [22,23,24]. When the IgG binding Z-domains are expressed on the OM of *E. coli* by autodisplay technology, the autodisplayed Z-domains exhibit the same orientation. By utilizing these autodisplayed Z-domains, the Fc region of capture antibodies is arranged on the autodisplayed Z-domains, allowing the capture antibodies to expose both antigen binding sites [12,21,25]. Unlike viruses, *E. coli* can self-proliferate and can be easily separated by centrifugation. Therefore, Z-domain autodisplaying *E. coli* cells may be applied as solid support for immunoassays [26,27]. Z-domain autodisplaying *E. coli* cells may be used to improve the sensitivity of immunoassays through orientation control of capture antibodies [25]. In addition, the OM of Z-domains autodisplaying *E. coli* cells can be isolated, and the OM layer can be formed on the surface of the transducer of the biosensor [28]. This signifies that an immunoaffinity layer with orientation control effect may be created by immobilizing capture antibodies on the surface of the biosensor, following coating with Z-domains autodisplaying OM [11,12,28]. Immunoaffinity layers based on Z-domains autodisplaying OM have improved immunosensor sensitivity through orientation control of antibodies [11,12].

For purposes of commercial medical diagnosis, the price of a biosensor is an important consideration so various chemical, thermal, and electrochemical methods have been used to regenerate capture antibody-immobilized immunoaffinity layers in immunosensors [29,30]. Among these, the chemical method which utilizes denaturation of protein structures via acid/base and/or detergents is widely used for such regeneration [31,32,33]. In order to regenerate biomolecule-immobilized surfaces of biosensors while ensuring minimum damage to immobilized biomolecules, an acid buffer using amino acid glycine is widely employed [2,34].

In this study, an orientation-controlled immunoaffinity layer was generated by capture antibody treatment of the SPR biosensor, following layering of the Z-domains autodisplaying OM. The objective of this study is the regeneration of the immunoaffinity layer with retaining the binding affinity. In case of previous work, glutaraldehyde was used for the fixation of antibodies and it cause the decrease of the binding results [11], so no chemical cross-linker was selected to avoid the decrease of binding activity of autodisplaying Z-domains. In addition, a regenerative immunoaffinity layer was developed by optimizing the regeneration conditions. To minimize damage to the OM layer containing autodisplayed Z-domains, phospholipids etc., pH of the acidic regeneration buffer (based on glycine) as well as the concentrations of added detergents, such as sodium dodecyl sulfate (SDS) and Tween-20, were optimized. The regenerative immunoassay based on Z-domains autodisplaying *E. coli* cells as solid support was confirmed using the optimized regeneration buffer. The OM of Z-domains autodisplaying *E. coli* cells was isolated and coated onto microplates. The regenerative immunoassay based on the OM layer was confirmed using optimized regeneration buffer. Following confirmation of the regenerative immunoassay, the immunoaffinity layer was applied using the Z-domains autodisplaying OM on the surface of the SPR biosensor. The regenerative biosensing assay was tested and confirmed using the regeneration buffer.

## 2. Materials and Methods

### 2.1. Reagent

Horseradish peroxidase (HRP), C-reactive protein (CRP), human IgG (hIgG), Tween-20, glycine, Isopropyl β-d-1-thiogalactopyranoside (IPTG), ethylenediaminetetraacetic acid (EDTA), SDS and hydrochloric acid were obtained from Merck (Darmstadt, Germany). Rabbit polyclonal anti-HRP antibodies, rabbit polyclonal anti-CRP antibodies, fluorescein isothiocyanate conjugated goat polyclonal anti-CRP (anti-CRP(FITC)) antibodies, and fluorescein isothiocyanate labeled goat polyclonal anti-human (anti-human(FITC)) antibodies were purchased from Abcam (Cambridge, UK). Phosphate buffered saline (PBS) was obtained from LPS solution (Daejeon, Korea) and used as antibody dissolving buffer. In addition, 96-well microplates were purchased from SPL Life Science (Pocheon, Korea). The 3,3′,5,5′-Tetramethylbenzidine (TMB) substrate kit was obtained from Thermo Fisher Scientific (Waltham, MA, USA).

### 2.2. Culture of E. coli Cells with Autodisplayed Z-Domains and the Isolation of the OM

Z-domains autodisplaying vector, pET-Z-18-3, was constructed by cloning the Z-domain, which was a PCR-amplified, engineered peptide from the B-domain of protein A in *S. aureus*, as described in previous studies [11,12,13,25]. Z-domain autodisplaying pET-Z-18-3 plasmids were transfected into *E. coli* cells (BL21(DE3)) via electroporation. The transfected *E. coli* cells were grown overnight at 37 °C in Luria-Bertani (LB) broth with the antibiotic, ampicillin. Subsequently, *E. coli* cells, cultured overnight, were washed and diluted 100-fold in freshly prepared LB broth with antibiotics. *E. coli* cells were grown at 37 °C with vigorous shaking until the optical density (OD) reached 0.7 at a wavelength of 600 nm. For the induction of autodisplayed Z-domains, 1 mM IPTG was added to the *E. coli* cells, which were cultured at 30 °C for 1 h. After induction, *E. coli* cells were harvested and washed thrice with PBS. The OM of Z-domains autodisplaying *E. coli* cells was isolated according to previous studies [28]. In brief, the rigid peptidoglycan layer, of *E. coli* cell walls was hydrolyzed by lysozyme treatment (200 μg/mL), resulting in the formation of a spheroplast. To assist with lysozyme penetration into the periplasm, 20 mM sucrose and 0.2 mM EDTA were added to enhance osmosis and control the surface charge. Following spheroplast formation, the OM of *E. coli* cells was isolated as a liposome by the addition of 2% Triton X-100 solution with 10 mM MgCl2 in 50 mM Tris-HCl pH 8.0. Following purification of OM particles by sequential centrifugation steps at 20,000 rpm, the isolated OM was resuspended with PBS.

### 2.3. Regenerative Assays Based on E. coli Cells and Their Outer Membrane

For *E. coli* cells-based regenerative assay, Z-domains autodisplaying *E. coli* cells were resuspended with the OD600 value of 1.0 PBS. Following, anti-HRP antibodies at a concentration of 1.0 μg/mL in PBS were added to 100 μL of Z-domains autodisplaying *E. coli* cells. Following incubation for 1 h, *E. coli* cells were washed thrice in PBS. The analyte, 10-ng/mL HRP sample, was added, and *E. coli* cells were incubated for 1 h. Following the washing steps, 100 μL of regeneration buffer was added to analyte-bound *E. coli* cells for 2 min. After regeneration, *E. coli* cells were washed three times and resuspended with 100 μL of PBS. These regenerated *E. coli* cells were used as a new solid support for the HRP immunoassay. The *E. coli* cells-based immunoassay was repeated four times. For the quantification of the bound HRP, the chromogenic reaction of the TMB solution was used. 100 μL of TMB solution was treated to the *E. coli* cells for 30 min, and the reaction was quenched with 2 M sulfuric acid solution. After then, the OD value was measured at the wavelength of 450 nm by using a microplate reader (Molecular Devices, San Jose, CA, USA).

For the OM-based regenerative assay, 300 μg/mL of isolated OM was added to the 96-well microplate and incubated for 2 h to form the OM layer on the surface of the microplate. Following OM layer formation, the plate was washed thrice with PBS and treated with anti-HRP antibodies in PBS at a concentration of 1.0 μg/mL for 1 h. Subsequently, the microplate was washed thrice, and treated with 10 ng/mL HRP samples for 1 h. Following the washing steps, 100 μL of regeneration buffer was added for 2 min, and eluted antibodies and antigens were immediately washed using PBS. The HRP immunoassay was repeated four times using the regenerated OM layer-coated microplate. For the quantification of the bound HRP, the TMB solution was treated for 30 min. After quenching using sulfuric acid solution, the OD value was measured at the wavelength of 450 nm.

### 2.4. SPR Measurement

A commercial SPR biosensor system from Reichert Technologies Life Science (Buffalo, NY, USA) was used for this study. The bare gold chip was used for OM-based regenerative biosensing. The volume of the SPR fluidic chamber was 7 μL. For immunoaffinity layer formation on the surface of the gold chip, isolated OM solution at 300 μg/mL concentration was treated with a flow rate of 7 μL/min for 10 min. The remaining OM isolation was washed with PBS for 10 min. After OM layer formation, 1 μg/mL of anti-CRP antibodies in PBS was bound on autodisplayed Z-domains on the OM layer for 5 min. After washing, 1 μg/mL CRP sample was treated for 5 min. For regeneration of the OM immunoaffinity layer with autodisplayed Z-domains, the regenerative solution was treated for 2 min at a flow rate of 7 μg/mL. Following the regeneration step, CRP biosensing was repeated 4 times using the regenerated OM layer on the gold chip of the SPR biosensor. Captured CRP was quantified by subtraction of the SPR signal after CRP treatment from the SPR signal before CRP treatment. The regeneration rate was calculated by the comparing the SPR signal after OM layer formation with the SPR signal following regeneration.

## 3. Results and Discussion

### 3.1. Optimization of Regenerative Assays

The general methods used for the elution of specific protein-protein binding, included denaturing of protein structures or elution through competitive binding. In this case, additional denaturation step was required to eliminate competitors. Therefore, the denaturation of protein structures was used to form the regenerative immunoaffinity layer in this study. The Z-domain, which is an engineered peptide from the B-domain of protein A, has binding affinity to the Fc region of antibodies. Protein A has been widely used for antibody purification, and acidic buffer was used to elute the protein A-antibody complex in protein A resin-based affinity chromatography. From this reason, acidic conditions were test for the regeneration process. For the optimization of the regenerative condition of Z-domains autodisplaying OM layer, HRP was selected as the model analyte. Unlike other proteins, HRP can be detected and quantified directly by the TMB or luminol reaction with hydrogen peroxide. After the optimization of the regenerative conditions, a regenerative immunoaffinity layer was tested using the SPR biosensor. SPR biosensor is a non-labeling optical sensor, which can detect the analyte solely via the capture antibody layer (immunoaffinity layer) without depending on detection antibodies.

**Figure 1:**
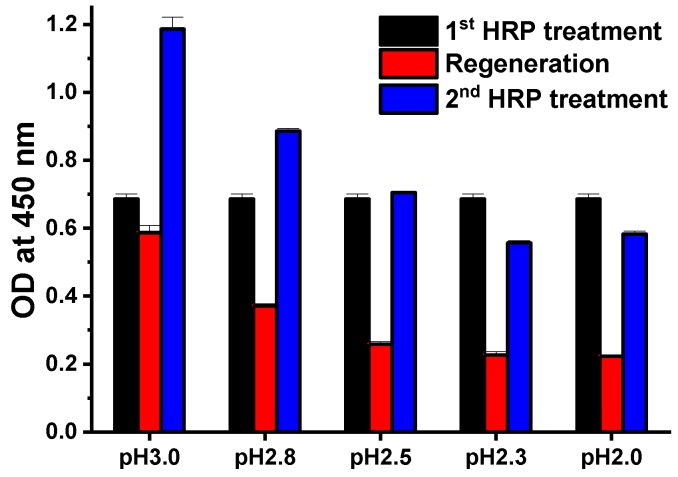
The optimization of the acidic conditions.

To optimized acidic conditions for the regeneration process, 0.1 M glycine buffer was tested at various pH levels ranging from 2.0 to 3.0 (Figure 1). For the capture antibodies, 1 μg/mL of anti-HRP antibodies was added to Z-domain autodisplaying *E. coli*, which were treated with 10 ng/mL HRP. Following formation of the Z-domain-anti-HRP-HRP complex, glycine buffers of various pH levels were added for 5 min. After the regeneration step, anti-HRP and HRP were added under the same conditions for the subsequent immunoassay. HRP bound on Z-domains autodisplaying *E. coli* was quantified by measuring OD at a wavelength of 450 nm following the TMB reaction. Compared to the OD value of the first HRP treatment (black), all OD values decreased after regeneration (red), and continued to decrease as the pH of the buffer decreased. This suggests that as acidity increased, the degree of Z-domains and antibody denaturation became more severe, resulting in a disruption in the binding between autodisplayed Z-domains and capture antibodies. OD values after regeneration (red) wasn’t near zero even all or nearly all of the HRP was eluted. It resulted from the autooxidation of TMB and from light scattering of *E. coli* cells in the reaction solution. Following regeneration, when anti-HRP and HRP were added a second time, the OD of all tests (blue) increased. This suggests that anti-HRP and HRP complex were eluted, and that treated anti-HRP and HRP formed a complex with regenerated autodisplayed Z-domains. At high pH (i.e., pH 3.0 and pH 2.8), the result of the second HRP treatment (blue) was increased relative to the first treatment (black) and showed high OD values following regeneration (red). This suggests that regeneration was not complete at high pH. Therefore, the HRP immunocomplex remained bound to the autodisplayed Z-domains. In contrast, in low pH (i.e., pH 2.3 or pH 2.0), the result of a second HRP treatment (blue) decreased relative to the first treatment (black). This suggests that strong acidic conditions damaged the solid support of the immunoassay, *E. coli* cells and autodisplayed Z-domains. These results, indicated that the optimal pH for stable regeneration without damage to autodisplayed Z-domains, was 2.5.

**Figure 2:**
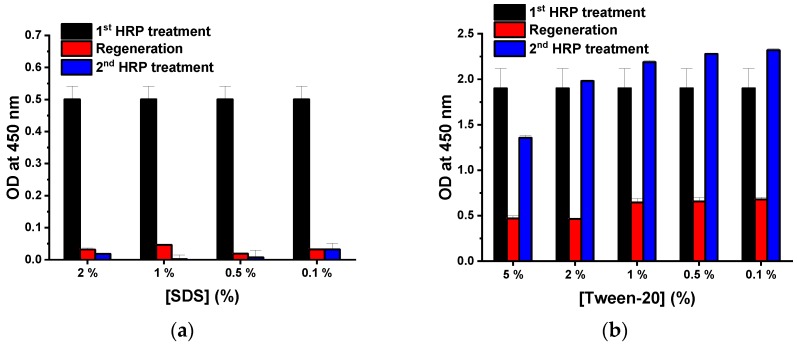
Test of additive detergents, (**a**) SDS and (**b**) tween-20.

**Figure 3:**
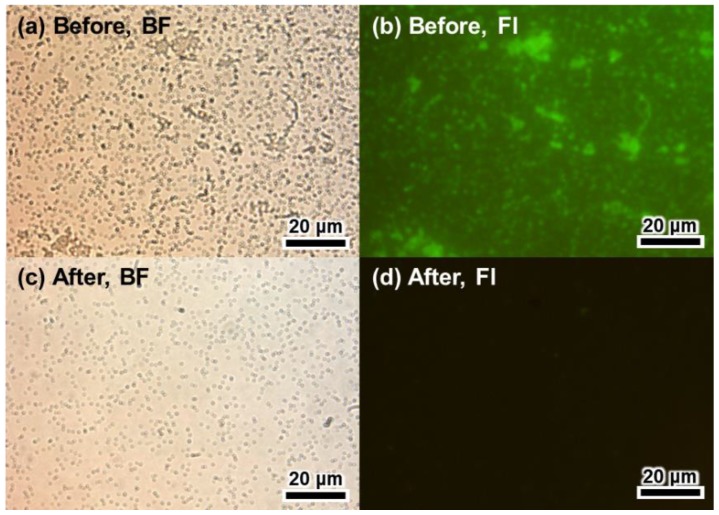
Microscopic images of Z-domains autodisplaying *E. coli* with antibody labeling before and after regeneration. (**a**) Bright field and (**b**) fluorescence images before regeneration and (**c**) bright field and (**d**) fluorescence images after regeneration.

In general, the regeneration solution or the stripping solution used for antibody elution had detergent added to maximize performance. For the better performance of the regeneration, various concentrations of detergents were test. SDS is an anionic surfactant that can denature proteins into their primary structures. Therefore, the regeneration performance of the glycine buffer was tested at various SDS concentrations. At all SDS concentrations, the OD values after regeneration (red) were less than 10% of those before regeneration (black) (Figure 2a). OD values did not increase with the second treatment of anti-HRP and HRP. In addition, the pellet size of *E. coli* cells during the washing step was significantly decreased after treatment with the SDS regeneration buffer. This suggests that a strong detergent, such as SDS, may seriously damage *E. coli* cells and autodisplayed Z-domains on its OM. Therefore, the amount and the antibody immobilizing ability of solid supports were decreased in the *E. coli* cell-based immunoassay. To reduce damage to *E. coli* cells and to increase regenerating ability of the buffer, Tween-20 (a widely used detergent in immunoassays) was selected. Various concentrations of Tween-20 (0.1%, 0.5%, 1%, 2%, and 5%) added to glycine buffer were tested at pH 2.5 (Figure 2b). In comparison with the first HRP immunoassay (black), OD values decreased after regeneration (red), and the OD values increased after the second HRP immunoassay. For 2% and 5% Tween-20, OD values after regeneration (red) were less than 35% of those before regeneration (black). Differences between the OD values of the first HRP immunoassay and the second HRP immunoassay were minimal (less than 5%) at a concentration of 2% Tween-20 containing glycine buffer at pH 2.5. However, in the second HRP immunoassay, the OD value corresponding to 5% Tween-20, decreased by 28.4%. It appears that a high concentration of Tween-20 may also damage *E. coli* cells [35]. Conversely, less than 2% of Tween-20 samples showed OD values which increased by more than 13% in the second HRP immunoassay. This suggests that regeneration was not complete. Therefore, it may be surmised that the anti-HRP and HRP complex of the first immunoassay was not eluted perfectly during the regeneration step. The regeneration effect of optimized conditions was tested by fluorescence microscopy. Briefly, 3 μg/mL of hIgG was immobilized on the Z-domains autodisplaying *E. coli* cells for 1 h, and 3 μg/mL of anti-human(FITC) antibodies were treated for 1 h. hIgG-anti-human(FITC) immunocomplex bound Z-domains autodisplaying *E. coli* were observed by fluorescence microscopy before and after regeneration. Clear fluorescence images of hIgG-anti-human(FITC) immunocomplex immobilized Z-domains autodisplaying *E. coli* were observed before regeneration (Figure 3b). In contrast, the fluorescence signal disappeared following the regeneration step (Figure 3d). This suggests that the hIgG-anti-human(FITC) immunocomplex was eluted by the regeneration step. These findings confirmed that 2% Tween-20 containing 0.1 M glycine buffer at pH 2.5 represented the optimal conditions for regeneration.

### 3.2. Regenerative Immunoassay Based on Z-Domains Autodisplaying E. coli

**Figure 4:**
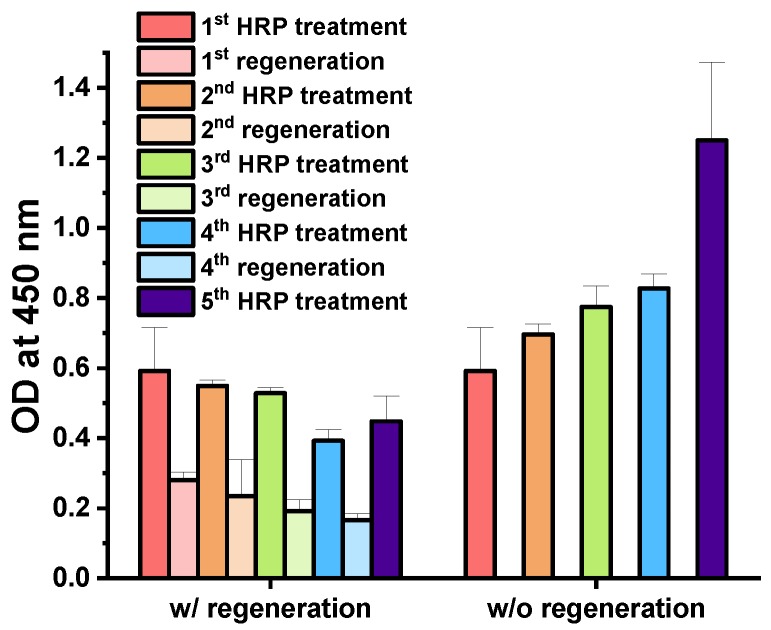
Regenerative immunoassay based on Z-domains autodisplaying *E. coli* cells.

The optimized regeneration conditions were tested via sequential regenerative immunoassays. First, the regenerative immunoassay based on the *E. coli* cells was tested. Then, the regenerative immunoassay using affinity layer based on Z-domains autodisplaying OM was test. For the *E. coli* based immunoassay, Z-domains autodisplaying *E. coli* cells were used as the solid support of the immunoassay, and HRP was selected as the model analyte. Following the HRP immunoassay, the Z-domains autodisplaying *E. coli* cells were regenerated by treatment of 2% Tween-20 in 0.1 M glycine buffer at pH 2.5. After regeneration, the HRP immunoassay was repeated. The regenerative immunoassay based on the Z-domains autodisplaying *E. coli* cells, the regeneration steps were repeated 4 times, and the HRP immunoassays were repeated five times. For the comparison group, an *E. coli* cells-based immunoassay was repeated five times without any regeneration steps; all assays were repeated in triplicate. OD values of the second, third, fourth, and fifth HRP immunoassays decreased by 7.3%, 10.0%, 33.7%, and 24.3% in comparison with the first HRP immunoassay, respectively (Figure 4). The *p*-values of Student’s *t*-tests between subsequent HRP immunoassays and the first HRP immunoassay were 0.58, 0.42, 0.05, and 0.15, respectively. All *p*-values were higher than 0.05; hence, indicating the absence of statistically significant differences between HRP immunoassays during regeneration. In comparison with the OD values following the first regeneration, OD values of the second, third, and fourth regeneration decreased by 16.2%, 12.9%, and 28.4%, respectively. Therefore, in the case of autodisplaying *E. coli* cells-based immunoassay, the regenerative immunoassay was validated with less than 10% deviation until the third HRP treatment. The OD values of the second, third, fourth, and fifth HRP immunoassays without regeneration increased by 7.6%, 30.7%, 39.7%, and 111.1% in comparison with the first HRP immunoassay, respectively. The *p*-values for the Student’s *t*-tests between subsequent HRP immunoassays and the first HRP immunoassay were 0.57, 0.08, 0.03, and 0.01, respectively. Thus, following the fourth HRP immunoassay without regeneration, the results were statistically significant compared to the initial result of the immunoassay. This suggests that continuous treatment of anti-HRP and HRP bound to autodisplayed Z-domains on *E. coli* cells without elution may cause consequential increases of OD values in the HRP immunoassays. These data support the elution effect between immunocomplex and autodisplayed Z-domains by the regeneration step. The decrease in OD values of immunoassays and regeneration may be explained by the repeated centrifugation steps which required repeated changing of the buffer and washing of the Z-domains autodisplaying *E. coli* cells. Repetitive processes of spin down and resuspension of *E. coli* cells results in cell loss. Thus, repetitive *E. coli* cells-based immunoassays and regeneration steps may lead to decreasing OD values due to loss of solid support.

**Figure 5:**
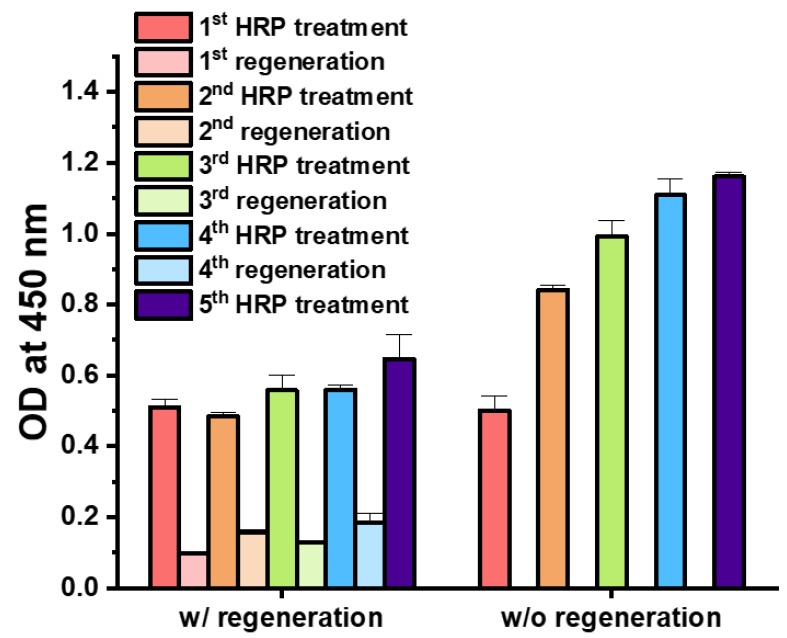
Regenerative immunoassay based on Z-domains autodisplaying OM layer.

To overcome any signal drop in *E. coli* cells-based immunoassays during the centrifugation steps, OM of Z-domains autodisplaying *E. coli* cells was isolated, and the surface of the microplate was coated with isolated OM. The OM layer coating modified the surface of the microplate into IgG-binding autodisplayed Z-domains. Subsequently, the immunoaffinity layer was formed by treatment with the capture antibodies, anti-HRP. When the OM layer-coated microplate was utilized as the immunoaffinity layer, an extra centrifugation step for washing or buffer change was not required. Therefore, it may prevent the signal from decreasing and the loss of solid support. Following formation of the OM-based immunoaffinity layer, 10 ng/mL of HRP was added for 1 h and quantified by the colorimetric measurement of OD at 450 nm wavelength after the TMB reaction. To test the regenerative immunoassay based on the OM of the Z-domains autodisplaying *E. coli*, the regeneration steps were repeated four times, and the HRP immunoassay were repeated five times. For the comparison group, an OM-based immunoassay was repeated five times without any regeneration steps; all assays were repeated in triplicate. OD values of the second HRP immunoassays decreased by 5.1% and those of the third, fourth, and fifth HRP immunoassays increased by 9.5%, 9.8%, and 24.5% compared to the first HRP immunoassay, respectively (Figure 5). The *p*-values of the Student’s *t*-tests between subsequent HRP immunoassays and the first HRP immunoassay were 0.14, 0.15, 0.14, and 0.08, respectively. This suggests that all 5 HRP immunoassays were not significantly different. Besides, the OD values following regeneration decreased by less than 20%. OD values of the second, third, fourth, and fifth HRP immunoassays without regeneration increased by 65.0%, 94.4%, 117.4%, and 127.6% in comparison with the first HRP immunoassay, respectively. In addition, *p*-values of the Student’s *t*-test between the subsequent HRP immunoassays and first HRP immunoassay were less than 0.0001, in all cases. Thus, in the absence of the regeneration step, HRP immunoassay results were significantly different from the initial result of the immunoassay. In the OM-based immunoassay, similar to the *E. coli* cells-based immunoassay, continuous treatment of anti-HRP and HRP bound to the Z-domains autodisplaying immunoaffinity layer without elution caused a significant increase of OD values in the HRP immunoassays. In addition, in the OM-based immunoassay, there was no loss of solid support due to centrifugation steps, and thus, signal strength was considerably stronger. These data support the elution effect between antibody-antigen immunocomplex and Z-domains autodisplaying OM layer by optimized regeneration buffer. From these results, in the case of the Z-domains autodisplaying OM-based immunoassay, the regenerative immunoassay was verified to be stable and possible with less than 10% deviation following 4 repetitions.

### 3.3. Regenerative Immunoaffinity Layer for SPR Biosensor

**Figure 6:**
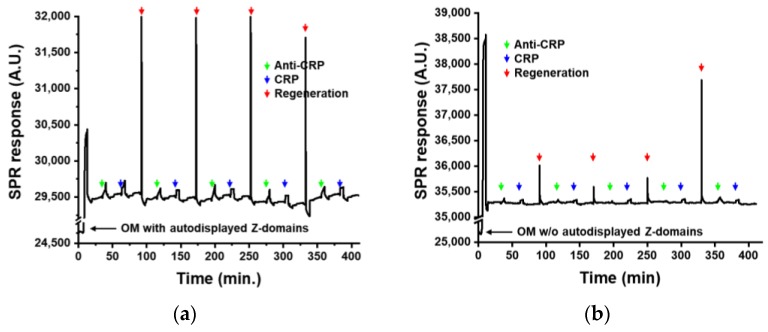
Regenerative immunoaffinity layer for SPR biosensors based on (**a**) OM with autodisplayed Z-domains and (**b**) OM without any autodisplayed target.

The Z-domains autodisplaying OM layer was applied to the immunoaffinity layer of SPR biosensor. The SPR biosensor is an evanescence field-based optical biosensor, and the thickness of the evanescence field of the SPR biosensor is less than 100 nm. This suggests that the immunoaffinity layer should be thinner than 100 nm for measurements using the SPR biosensor. The OM of Z-domains autodisplaying *E. coli* was isolated as a liposome using lysozyme and Triton X-100 and layered on a two-dimensional gold substrate (the transducer of the SPR biosensor). This OM layer has a thickness of 2 nm, which is sufficiently fine for SPR measurement. The OM of *E. coli* has a lipopolysaccharide-connected phospholipid bilayer structure. In the inner layer of the OM, lipoproteins are attached to peptidoglycan in a manner which leaves a space in between. Following isolation as a liposome, outer surface of the OM contains autodisplayed Z-domains and hydrophilic lipopolysaccharides, while the inner surface contains lipoproteins. When the isolated OM forms a layer on a hydrophobic substrate, such as gold, the hydrophobic inner surface of the OM contacts the hydrophobic substrate, and autodisplayed Z-domains are exposed on the surface. Thus, an immunoaffinity layer with orientation control may be developed using the OM layer with autodisplayed Z-domains. In this study, a regenerative immunoaffinity layer based on the Z-domains autodisplaying OM layer was tested by the SPR biosensor. Acute phase inflammatory CRP was selected as the analyte. CRP has been widely used as a biomarker in the diagnosis of inflammatory diseases, such as osteoarthritis, rheumatoid arthritis, infections, vasculitis, tumors, and others. Following formation of the OM layer on the surface of the SPR biosensor, anti-CRP antibodies were added for 5 min. Subsequently, CRP with a concentration of 1 μg/mL was added. After CRP measurement, the SPR biosensor was regenerated with optimized buffer. After regeneration, anti-CRP and CRP were added for the assessment. The regeneration steps were repeated four times, and CRP biosensing was repeated five times. The OM layer without autodisplayed Z-domains was used as the comparison group. SPR response was monitored during the regeneration processes. Following OM injection, SPR response increased significantly (Figure 6). After the washing step, the SPR response generated by the OM layer formation was calculated to be 4708 A.U. OMs contain various proteins, lipopolysaccharides, and phospholipids, resulting in the SPR signal of the OM layer being significantly high. Following OM layer formation, anti-CRP antibodies, and CRP was added sequentially. Following CRP biosensing, immunoaffinity layer based on OM with autodisplayed Z-domains were regenerated. It was observed that, during CRP immunoassays, SPR response was increased and returned to a level close to the SPR response of the first OM layer formation after regeneration steps. In comparison with the SPR response of OM layer formation, the signal differences from four regeneration steps were calculated to be 33.03, 33.84, 69.54, and 14.88, respectively. These values were only 0.7%, 0.7%, 1.5%, and 0.3% of the SPR signal of the OM layer. This indicated that optimized regeneration conditions may efficiently and stably regenerate the immunoaffinity layer based on OM with autodisplayed Z-domains. In comparison with the SPR responses of anti-CRP treatment, the signal differences in each biosensing cycles after CRP treatments were calculated to be 236.71, 205.93, 226.43, 205.92, and 218.57, respectively. The repeatability of the SPR biosensing was calculated to be a coefficient of variation of 6.09%. It means that the regeneration of the immunoaffinity layer shows a good repeatability. From these results, the immunoaffinity layer based on OM with autodisplayed Z-domains was successfully regenerated with retained antibody binding affinity. With regard to the OM layer without autodisplayed Z-domains, an increase in the SPR signal after anti-CRP and CRP treatment was not detected, indicating the absence of non-specific binding of antibodies or analytes on the OM layer of the SPR biosensor. Such reduced non-specific binding may reduce noise and increase the reliability of SPR biosensing. These results confirmed that the regenerative immunoaffinity layer based on OM with autodisplayed Z-domains had less than 2% of variations during five CPR biosensing procedures. This enabled four times of regeneration could save four extra SPR chips, time for the immunoaffinity layer formation, and the additional resource such as the isolated OM particles. SPR chip costs normally 50 dollars so this regenerative immunoaffinity layer could reduce at least 200 dollars for biosensing five times. It was confirmed that the sensitivity of SPR biosensor was improved by the orientation control effect of autodisplayed Z-domains [11,12]. In addition to this orientation control effect, the regenerative immunoaffinity layer based OM with autodisplayed Z-domains may enable stable and sensitive biosensing at a reduced cost and also prevent valuable resources such as rare metals from being wasted.

## 4. Conclusions

The findings of this study indicated that the immunoaffinity layer based on Z-domains autodisplaying OM with orientation control of antibodies was regenerative. Since the developed immunoaffinity layer consists of biomaterials, such as proteins and phospholipids, glycine buffer was selected as the regeneration buffer to optimize regeneration conditions. Following tests of various pH conditions, 0.1 M glycine buffer with the pH value of 2.5 was selected. Subsequently, detergent was tested for more effective regeneration potential. Strong anionic SDS was confirmed to be too strong for use in the regeneration of proteins containing solid support. Therefore, 2% Tween-20 was optimized by testing at various concentrations. These optimized regeneration conditions were tested using Z-domains autodisplaying *E. coli*. *E. coli* cells were used as solid supports, and the regenerative effect of the HRP was tested via immunoassay. The results confirmed that 3 sequential regenerative immunoassays were feasible with less than 10% variation. To overcome the loss of *E. coli* cells by centrifugation, the OM with autodisplayed Z-domains-based HRP immunoassay was performed for the regeneration effect. Z-domains autodisplaying OM-based HRP immunoassay was confirmed as feasible following 4 repetitions with less than 10% variation. The Z-domains autodisplaying OM was applied to the SPR biosensor as an immunoaffinity layer. The regenerative immunoaffinity layer with autodisplayed Z-domains was tested using CRP and validated following five repeated measurements of regeneration. The results confirmed that regenerative immunoaffinity based on OM with autodisplayed Z-domains had less than 2% of variation during four regeneration processes. In addition, the repeatability of five times of CRP biosensing was calculated to be 6.09%. This regenerative immunoaffinity layer can be regenerated without any reduction of antibody binding affinity. It means that orientation controlled antibody layer can be easily reconstituted for further immunoassays or biosensings. Therefore, the regenerative immunoaffinity layer may reduce the total cost of medical diagnosis and conserve the use of novel metals, such as gold. Thus, this regenerative technology of the Z-domains autodisplaying OM-based immunoaffinity layer may be applied to the surface of various immunosensors to improve their sensitivity through orientation control of antibodies. It can also reduce diagnostic costs by via the reusing of transducers and immunoaffinity layers.
